# Non-Invasive Prenatal Diagnosis of Retinoblastoma Inheritance by Combined Targeted Sequencing Strategies

**DOI:** 10.3390/jcm9113517

**Published:** 2020-10-30

**Authors:** Amy Gerrish, Benjamin Bowns, Chipo Mashayamombe-Wolfgarten, Elizabeth Young, Samantha Court, Joshua Bott, Maureen McCalla, Simon Ramsden, Michael Parks, David Goudie, Sue Carless, Samuel Clokie, Trevor Cole, Stephanie Allen

**Affiliations:** 1 West Midlands Regional Genetics Service, Birmingham Women’s and Children’s NHS Foundation Trust, Birmingham B15 2TG, UK; benjamin.bowns@nhs.net (B.B.); chipo.m-wolfgarten@nhs.net (C.M.-W); elizabeth.young6@nhs.net (E.Y.); samantha.court2@nhs.net (S.C.); joshua.bott@nhs.net (J.B.); m.mccalla@nhs.net (M.M.); sue.carless1@nhs.net (S.C.); s.clokie@nhs.net (S.C.); trevor.cole1@nhs.net (T.C.); stephanie.allen13@nhs.net (S.A.); 2 Manchester Centre For Genomic Medicine, University of Manchester, St Mary’s Hospital, Manchester Academic Health Science Centre, Manchester M13 9WL, UK; simon.ramsden@cmft.nhs.uk; 3 Nonacus Limted, Birmingham Research Park, Birmingham B15 2SQ, UK; michael.parks@nonacus.com; 4 East of Scotland Regional Genetics Service, Ninewells Hospital and Medical School, Dundee DD1 9SY, UK; david.goudie@nhs.scot

**Keywords:** retinoblastoma, prenatal diagnosis, non-invasive, cell-free DNA

## Abstract

Retinoblastoma, the most common childhood eye cancer, presents in two forms: heritable or sporadic. Heritable retinoblastoma is caused by a germline mutation in the *RB1* gene. Early diagnosis of children at risk of inheriting an *RB1* mutation is crucial to achieve optimal clinical outcome. Currently, the majority of genetic testing is performed on newborns, which has multiple disadvantages for both families and the healthcare system. We have developed a non-invasive prenatal diagnosis (NIPD) service for retinoblastoma, available from 8 weeks’ gestation, which uses a combination of massively parallel sequencing (MPS) techniques, dependent on the inheritance model. Detection of paternal or suspected de novo *RB1* variants is achieved through amplicon-based MPS. NIPD of a fetus at risk of maternal inheritance is performed using capture-based targeted sequencing and relative haplotype dosage analysis. In addition, we show proof of principle of how capture-based sequencing can be used for de novo variants unsuitable for amplicon-based testing. In total, we report the NIPD of 15 pregnancies, results of which show 100% concordance with all postnatal testing performed at the time of publication (*n* = 12) with remaining pregnancies ongoing. NIPD of retinoblastoma therefore offers a viable alternative to newborn genetic testing.

## 1. Introduction

Retinoblastoma (Rb) is the most common childhood eye cancer with an incidence of ~1:15,000–1:20,000 live births (reviewed in [1]). Over 99% of retinoblastomas are due to bi-allelic inactivation in the *RB1* gene, caused by either two somatic mutations or an initial germline mutation followed by a subsequent somatic hit as described in Knudson’s two-hit hypothesis [2]. Approximately 40% of Rb cases carry a germline mutation and can be further classified as either familial, where the patient has inherited a predisposition to Rb from an affected parent, or sporadic heritable, where there is no family history and Rb has resulted from a de novo pre-zygotic germline mutation [3]. A child is at 50% risk of inheriting an *RB1* mutation if either of their parents carry a germline *RB1* variant. Furthermore, siblings of a patient with a de novo *RB1* mutation have an approximate 1% risk of also carrying the mutation due to germ cell mosaicism [4]. Where a family history is known, early diagnosis is paramount to achieve optimal clinical outcome with the least treatment morbidity. Currently, the majority of testing is performed on postnatal cord blood, with the aim of achieving a diagnostic result within 14 days of birth. This tight timeline can pose challenges to both the healthcare system and parents. It requires considerable co-ordination of the antenatal, paediatric, genetic laboratory and retinoblastoma services. Even within a nationalised health service, this is not easy to achieve and is further challenged in couples not wishing hospital deliveries. The laboratory costs are also increased by having to run the analysis as an urgent test. For parents undergoing predictive genetic testing, this is an additional psychological burden at a time when bonding with a new baby is important and individuals may feel especially vulnerable. Furthermore, many families may live several hours away from their retinoblastoma service providers and have to arrange, and travel to, a precautionary early appointment in case the results are delayed.

A current alternative to newborn screening is prenatal testing, either by chorionic villi sampling (CVS) at 11 weeks’ gestation or amniocentesis at 16 weeks. However, a recent Dutch review reports only 12% of parents with an Rb family history take up this service [5] and our experience suggests this number is even lower in the UK. This is likely partly due to the invasive nature of the test, which includes a surgical procedure with an often-reported 0.5% chance of miscarriage [6].

Non-invasive prenatal diagnosis (NIPD), which analyses cell-free fetal DNA within the mother’s blood, offers all the potential advantages of a prenatal result while avoiding the issues associated with an invasive procedure. Unlike non-invasive prenatal testing (NIPT) screening, NIPD is considered a definitive diagnostic test, where confirmation by invasive means is not required [7]. Furthermore, NIPD testing is available from 8 weeks’ gestation, earlier than both CVS and amniocentesis. If a positive genetic result is obtained, an Rb management plan could be put in place prior to birth. Parents would be able to come to terms with the diagnosis in a timely manner before the arrival of their child. A negative result would give peace of mind to the parents and could mean a child avoids unnecessary neonatal ophthalmic checks.

While there have been several reports in the literature showing case studies and proof of principles for NIPD assays for single gene disorders (reviewed in [8]), there has been relatively little clinical implementation. The majority of NIPD services that have been developed are designed to detect paternal or de novo variants [9,10] as the detection of maternally inherited alleles is more challenging due to the presence of cell-free DNA (cfDNA) of maternal origin in maternal plasma. We previously developed NIPD for X-linked [11] and autosomal recessive disorders [12] which allows both maternal and paternal inheritance to be determined by relative haplotype dosage (RHDO) analysis. This method overcomes the difficulty in detecting maternal variants and, as it uses linkage-based approach, does not require test development for family-specific variants, some of which may be unsuitable for direct detection. These tests are now part of our routine clinical service in the UK, providing non-invasive prenatal diagnosis for pregnancies at risk of cystic fibrosis (CF), spinal muscular atrophy (SMA), Duchenne muscular dystrophy (DMD) or Becker muscular dystrophy (BMD), with over 150 pregnancies tested since September 2016 [7].

Here, we outline the development and implementation of clinical NIPD testing for retinoblastoma. We describe a comprehensive, cost-effective NIPD service available to families with a positive family history of the disease, which uses a combined assay approach. Paternally inherited and likely de novo mutations are detected directly using amplicon-based massively parallel sequencing (MPS), whereas NIPD of maternally inherited mutations uses a capture-based targeted MPS approach and haplotype analysis. In addition, we also show how capture-based targeted MPS can be adapted for the direct detection of larger paternal or likely de novo mutations, which would not be possible using an amplicon-based test.

## 2. Material and Methods

### 2.1. Patient Samples

Initial recruitment and validation samples were collected as part of the NIPSIGEN study (“NIPSIGEN: clinical translation of NIPD for SGDs”; REC approval number: 13/NW/0580). The delivery of NIPD by RHDO or direct amplicon-based mutation analysis is now part of routine clinical service delivered for the UK population by the West Midlands Regional Genetic service. Subsequent families described in this series provided consent for prenatal genetic testing in line with standard clinical practice. Plasma from non-pregnant healthy donors was also collected as part of the NIPSIGEN study.

To be eligible for testing, families needed to have a known family history of retinoblastoma, with a confirmed molecular diagnosis and appropriate reference samples (maternal and paternal or maternal, paternal and previous child) available. Samples were accepted for analysis from pregnant patients with singleton pregnancies at >8 weeks of gestation, confirmed by a scan.

### 2.2. Sample Processing and DNA Extraction

Maternal and control plasma was isolated from 10 mL blood samples received in Streck (La Vista, USA) BCT tubes and stored at −80 °C. Cell-free DNA was extracted either from 4 mL of plasma using the QIAsymphony Circulating Nucleic Acid kit (Qiagen, Hilden, Germany) into a final volume of 60 μL AVE buffer or from 2 mL plasma using a QIAamp DSP MiniElute virus kit (Qiagen, Hilden, Germany) into a final volume of 50 μL AVE buffer. Genomic DNA (gDNA) was extracted from leukocytes by QIAsymphony DSP DNA kit (Qiagen, Hilden, Germany).

### 2.3. Amplicon-Based MPS

Custom primers for each mutation were designed using Primer Quest Tool (IDT, Coralville, USA). Illumina sequence tags were then added. Primer validation was performed using a maternal gDNA sample plus gDNA from the affected family member (paternal or previous affected child) and control cfDNA. NIPD testing for a variant was offered following successful validation where the following conditions had been met: (1) mutant allele detected in the affected family member at the expected frequency, and (2) absence of the mutant allele in maternal gDNA and control cfDNA. In total, nine primer sets were designed and validated (Supplementary Table S1), eight of which were then subsequently used for NIPD testing.

NIPD testing comprised of sequencing maternal cfDNA in duplicate plus maternal gDNA, paternal gDNA and gDNA from a previous affected child where applicable. Five cfDNA samples from non-pregnant healthy individuals were also included as controls. Fetal fraction was determined by amplifying an additional maternal cfDNA sample using a primer pool targeting 42 informative single nucleotide polymorphisms (SNPs) across the genome (Supplementary Table S2).

Polymerase chain reactions (PCRs) were carried out using 20 μL (~3–9 ng) cfDNA or 10 ng gDNA plus 10 μL of Multiplex PCR mastermix (Qiagen, Hilden, Germany) and 200 nM of each primer in a final reaction volume of 20 μL. For PCRs using the fetal fraction primer pool, 10 μL of Multiplex PCR *Plus* mastermix (Qiagen, Hilden, Germany) was used. Cycling conditions were 95 °C for 5 min, followed by 30 cycles of 95 °C for 30 s, 60 °C for 3 min and 72 °C for 30 s, followed by 68 °C for 30 min. Amplicons were purified using Zymo-Spin Columns (Zymo Research, Irvine, USA) according to the manufacture’s instructions. Purified PCR products were then amplified for a second time to incorporate individual 6 bp barcodes for each sample. Indexing PCRs were carried out using 2 μL of purified PCR product, 10 μL of Multiplex PCR mastermix (Qiagen, Hilden, Germany) plus 5 µM of barcoding primer in a final reaction volume of 20 μL. Cycling conditions were 98 °C for 5 min, followed by 15 cycles of 95 °C for 30 s, 62 °C for 30s and 72 °C for 1 min, followed by 72 °C for 5 min. Amplicons were purified for a second time using Zymo-Spin Columns (Zymo Research, Irvine, USA) and quantified using a Qubit dsDNA BR Assay Kit (Thermo Fisher Scientific, Waltham, USA). DNA libraries were then pooled and sequenced on a MiSeq (Illumina Inc., San Diego, USA) using 2 × 80 cycles paired-end settings.

### 2.4. Bioinformatic Analysis

FASTQ files obtained from the MiSeq were quality-trimmed using Trimmomatic (v0.32), removing all reads below a quality score threshold of 30. Reads were aligned to the human genome, hg19, using Bowtie2 (v2.2.3). Alignment files were pre-processed, and duplicates were removed using a combination of Picard tools (v1.97) and Samtools (v0.1.19). Genomic coordinates plus reference and alternative alleles of the variant of interest were obtained using Mutalyzer API and BioPython-Entrez, respectively. Allele counts were acquired using the Platypus (v0.8.1) variant caller.

Fetal fractions were calculated using allele ratios of 42 highly heterozygous SNPs amplified within the maternal cfDNA. SNPs with a minor allelic ratio 0.01–0.13, indicating fetal origin, were used to calculate the cell-free fetal fraction (cffDNA) using the formula

$$\mathrm{cffDNA}\%=\frac{2\times \mathrm{Allele}\;B}{\mathrm{Allele}\;A+\mathrm{Allele}\;B}\times 100$$

To determine if the fetus had inherited the wild-type or mutant *RB1* allele, mutant allele ratios were compared between the maternal cfDNA, maternal gDNA and control cfDNA. For an affected diagnosis to be issued, the mutant allele was required to be present within the maternal cfDNA at levels expected given the calculated fetal fraction and above background levels seen in the control cfDNA. Mutant allele reads below the expected level and below or equal to levels within the control cfDNA were diagnosed as unaffected.

### 2.5. Capture-Based Targeted MPS

Using criteria described previously [11], we selected 4094 SNPs from dbSNP (144) with a reported average heterozygosity of ≥40% across a 5 Mb region on chromosome 13 (Chr13: 46,500,000–51,500,000) containing the *RB1* gene (NM_000321, Chr13: 48,877,883-49,056,122, hg19). The selected SNP coordinates were then uploaded to the NimbleDesign software (Roche Molecular Systems, Pleasanton, USA) and capture probes were designed using the highest stringency parameters to minimise the possibility of non-specific capture. The overall captured area was 260 Kb and SNPs were evenly distributed between the centromeric (1533 SNPs) and telomeric (1238 SNPs) regions flanking the *RB1* gene.

Capture-based MPS was performed as previously described [11]. In brief, DNA libraries were prepared from 20 to 100 ng input DNA. Up to 12 samples, equivalent to three prenatal diagnoses plus control samples, were multiplexed per run and sequenced on the MiSeq sequencing platform using 2 × 80 cycles paired-end settings. Bioinformatic analysis included quality trimming of reads, alignment to genome build hg19, removal of duplicates and variant calling to obtain SNP counts.

### 2.6. Relative Haplotype Dosage Analysis

Relative haplotype dosage (RHDO) measures the allelic imbalance between two haplotypes in plasma cfDNA to determine which haplotype has been inherited by the fetus. Haplotype phasing is conducted through genotyping of SNPs within the genomic DNA of the mother, father and a previous child [13,14]. For NIPD testing of mothers known to carry an *RB1* mutation, RHDO analysis was performed on the maternal chromosome, as described previously [12]. In brief, RHDO was achieved by firstly identifying the haplotype linked with the wild-type/mutant allele by DNA sequencing highly heterozygous bi-allelic SNPs within the *RB1* region in the previous child (unaffected or affected). The genotypes of the same SNPs are then determined in maternal and paternal DNA samples to allow haplotype phasing and to identify the maternal haplotypes linked with the mutant and wild-type alleles.

To ascertain which haplotype has been inherited by the fetus, SNPs that are heterozygous in the mother and homozygous in the father are selected and allele counts are obtained from maternal cfDNA sequencing. These SNPs are split into two groups (alpha and beta) depending on the genotype of the previous child. For alpha SNPs, the paternal allele inherited by the proband is identical to the maternal mutant allele. In the case of beta SNPs, the proband paternal allele is the same as the maternal wild-type allele. Alpha and beta SNPs are analysed separately and grouped into maternal haplotype blocks of a minimum of 25 SNPs, with each block representing a statistically independent result. Fetal fraction for each plasma sample was determined using SNPs that are homozygous in both parents but for alternative SNP alleles, i.e., AA and BB, as described previously [11,12].

### 2.7. Confirmation Testing

Confirmation testing of all prenatal results was performed by Sanger sequencing of DNA extracted from a postnatal cord blood sample at the Manchester Centre for Genomic Medicine following their routine diagnostic protocol.

## 3. Results

### 3.1. Amplicon-Based MPS

NIPD for Rb was requested by six couples with paternal history of retinoblastoma and six couples with a previous child identified as carrying a suspected de novo *RB1* single nucleotide variant (SNV). Following successful validation, NIPD for Rb was performed on 12 pregnancies. Maternal blood was taken at an average of 21 weeks’ gestation (range 12–35). Cell-free DNA extracted from the maternal plasma was amplified alongside maternal gDNA, paternal gDNA and control cfDNA. Genomic DNA from the previous affected child was also included as a positive control where the identified germline mutation was likely de novo.

**Figure 1:**
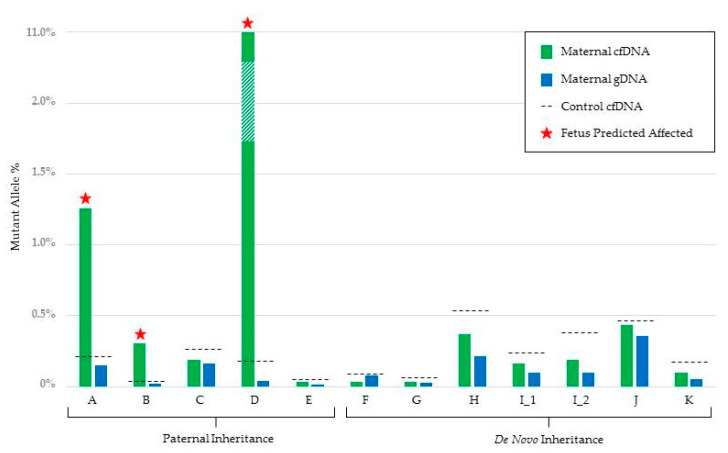
Graphical representation of the mutant allele % detected in both maternal cfDNA (green) and maternal gDNA (blue) for each family tested by amplicon-based MPS. The maternal cfDNA mutant allele % is an average of two duplicate samples. The maximum mutant allele % detected in the five control cfDNA samples is also shown (black dotted line). Tests reported as predicted to be affected are indicated with a star.

In couples where the father was affected with Rb, the paternal mutation was detected in the maternal cfDNA in three cases (Figure 1). In two families, the fetus was shown to be unaffected. One additional couple underwent successful NIPD validation but gave birth prior to full testing and newborn testing was performed in this instance.

All three fetuses shown to be gene carriers were delivered at term and early newborn assessment and treatment were expedited. Three of the six eyes had grade A tumours at first assessment and all retinoblastomas were treated solely with thermotherapy. At follow-up (2–5 years), one eye had a logMAR score of 0.14 and the remaining eyes had logMAR scores of 0.1 or better.

For the six unaffected couples, who had a previous child with a germline *RB1* mutation (total pregnancies = seven), all fetuses were found to be unaffected (Figure 1). Full results including read counts can be found in Table 1.

Fetal fraction ranged from 2.4 to 17.5%. Due to the direct nature of the test, a mutant allele could still be detected at fetal fraction <4%. However, it should be noted that a negative result in this instance would have been reported as inconclusive. All NIPD results have been confirmed where postnatal testing was available at the time of publication (n = 10). One case (Family A) who was part of the NIPSIGEN validation period had invasive prenatal testing which was concordant with the NIPD result. Where confirmation testing is not available, the pregnancies are ongoing.

### 3.2. Haplotype Analysis of Maternally Inherited Alleles

We performed NIPD by relative haplotype dosage for two pregnancies (Families L and M) at risk of maternally inherited *RB1* mutations.

**Figure 2:**
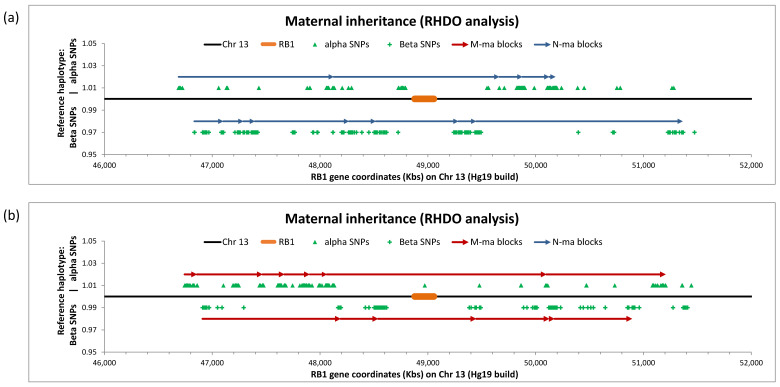
Graphic representation of NIPD by relative haplotype dosage analysis (RHDO) result for (**a**) Family L and (**b**) Family M at risk of maternal inheritance of retinoblastoma. Haplotype blocks are represented by contiguous arrows spanning a 5 Mb genomic region on chromosome 13 (black line) containing *RB1* (orange bar). Red arrows indicate the fetus has inherited the mutated maternal (M-ma) allele. Blue arrows indicate the fetus has inherited wild-type (N-ma) alleles. The position of informative single nucleotide polymorphisms (SNPs) used to identify haplotype blocks is shown for both alpha (green triangles) and beta (green crosses) SNPs.

The mother in Family L had a history of bilateral Rb (R*B1* c.120_21 delinsC p.Glu40Aspfs*25) as well as a previous child also affected with bilateral Rb. A maternal blood sample was taken during her second pregnancy at 29 weeks’ gestation for NIPD analysis. We observed a fetal fraction of 15.3%. After quality filtering, 401 (170 alpha and 231 beta) informative SNPs were identified, which formed five and eight haplotype blocks, respectively, across the region (Figure 2a). All blocks indicated the fetus had inherited the unaffected maternal chromosome. This was concordant with the postnatal molecular result, performed on the follow-up infant gDNA sample by Sanger sequencing.

The mother in Family M had a history of bilateral Rb (*RB1* c.1498 + 1 G > T) and a previous child who was also heterozygous for the mutation. A maternal blood sample was taken during her second pregnancy at 24 weeks’ gestation for NIPD analysis. We observed a fetal fraction of 14.11%. A total of 325 (175 alpha and 150 beta) informative SNPs were identified, which formed seven alpha and six beta haplotype blocks across the region (Figure 2b). In this analysis, all haplotype blocks indicated that the fetus had inherited the maternal chromosome carrying the *RB1* c.1498 + 1 G > T mutation and therefore would be affected. As of September 2020, this pregnancy is still ongoing and confirmation testing will be performed on postnatal cord blood.

### 3.3. NIPD of 2 Mb RB1 De Novo Deletion

Family N had previously had a child with bilateral Rb, which was shown by microarray analysis to have been caused by a deletion of approximately 2 Mb which incorporated the *RB1* gene (arr[hg19]13q14.2(47466483_49514186)x1). This deletion was not observed in the parents and therefore likely de novo. Due to the possibility of germline mosaicism, the couple requested NIPD when pregnant with their second child and a maternal blood sample was taken at 23 weeks’ gestation.

**Figure 3:**
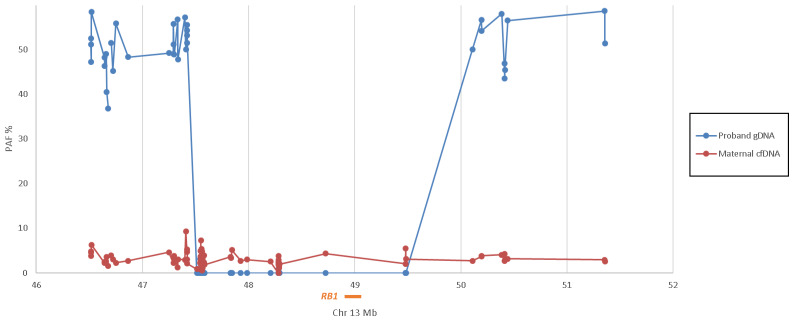
Graphic representation of the paternal allele frequency (PAF%) across the 5 Mb region of chromosome 13 containing *RB1* (orange bar) within the previous affected child (proband) gDNA (blue line) and maternal cfDNA (red line) of Family N. The affected child is hemizygous for chr13:47,515,019–49,482,240. The maternal cfDNA had a calculated fetal fraction of 6% and an average of 2.83% paternal alleles across the region, indicating that the fetus is unaffected.

Due to the size of the mutation, amplicon-based testing was not possible. We therefore performed capture-based MPS targeted to the 5 Mb *RB1* region. As with NIPD by RHDO, informative SNPs were genotyped within the maternal cfDNA, maternal and paternal gDNA plus genomic DNA of the child carrying the ~2 Mb deletion. SNPs which were AA in the mother and BB in the father (known as Type 1 SNPs [13]) were then selected across the region for further analysis. In normal inheritance, a child will be heterozygous AB for type 1 SNPs. In Figure 3, we show that the previous affected child of Family N is heterozygous (average paternal allele frequency = 50.62%) either side of the ~2 Mb deletion but homozygous for the maternal allele within this region (average paternal allele frequency = 0.21%), indicating that the deletion is located on the paternal chromosome.

Analysis of the cfDNA derived from maternal plasma detected a fetal fraction of 6.25%. Where the fetus is heterozygous for a type 1 SNP, we would therefore expect to detect the paternal allele at a frequency of ~3%. Paternal alleles were detected within the maternal cfDNA across the full 5 Mb region, including within the ~2 Mb region deleted in the previous affected child (average paternal allele frequency = 2.83%), indicating the fetus has inherited the full 5 Mb region from the father and was therefore predicted to be unaffected. This was confirmed through postnatal testing.

## 4. Discussion

Early diagnosis of retinoblastoma in individuals with a positive family history of the disease is important not only for patient management but for family information and wellbeing. Prenatal diagnosis of a fetus at risk offers significant benefits over newborn testing. As well as the potential for psychological and logistical advantages, studies have shown that intra-uterine and early postnatal retinoblastoma tumours grow rapidly and are more likely to compromise vision (reviewed in [3]). In some countries, ultrasonography and MRI may be performed if a fetal *RB1* mutation is identified prenatally. Furthermore, a study of 20 Canadian children with familial Rb found that scheduled early-term (36–38 weeks) delivery following prenatal detection of an *RB1* mutation led to an increase in several positive outcome measures including reduced invasive therapy and enhanced vision [15]. However, additional validation will be required to confirm whether improved visual outcomes are seen in a larger cohort, that treatment modality options are not restricted by gestation and that the known negative outcomes potentially associated with induction and early delivery do not outweigh these benefits.

Despite these possible advantages for patient management, current prenatal testing by CVS or amniocentesis is not an attractive option to parents, and the uptake is generally low. Since October 2016, we have offered NIPD testing to couples with a paternal history of Rb and to unaffected couples who have had a previous child affected with Rb, found to carry a likely de novo germline *RB1* mutation. NIPD for couples with a maternal history of retinoblastoma is more challenging due to excess cfDNA of maternal origin within maternal plasma. We have therefore developed NIPD for pregnant women affected with retinoblastoma using capture-based targeted sequencing and relative haplotype dosage. This paper describes the successful development and application of NIPD for Rb in 14 families (15 pregnancies) with a 100% concordance rate with current postnatal testing results (three pregnancies ongoing). Through the combination of amplicon- and capture-based targeted MPS, we are therefore able to provide cost-effective testing for paternal and de novo mutations as well as maternally inherited variants.

Testing is available from 8 weeks’ gestation and throughout pregnancy. For all NIPD testing, we stipulate a minimum gestational age of 8 weeks. This is to ensure the presence of adequate fetal-derived cfDNA within the maternal plasma. The fetal fraction is known to increase with maternal gestation [16,17]. In our recent publication evaluating our NIPD by RHDO service on over 150 pregnancies [7], less than 6% of tests required a second sample to be taken due to a low fetal fraction after 8 weeks’ gestation. In order to achieve a reportable result, we find that a fetal fraction >4% is often required for both amplicon- and capture-based NIPD, although as Family A shows, it is possible to detect the presence of a paternal or de novo mutation when the fetal load is less than this.

One limitation of NIPD for Rb testing is that, where haplotype analysis is involved, a previous child is required in order to phase the mutant haplotype. While the previous child does not have to be affected, this requirement still places limitations on the availability of the test. Proof-of-principle studies have demonstrated that micro-fluidics-based linked-read sequence technology can be used to deduce parental haplotypes directly without the need for reference samples [18,19], although the cost analysis for this currently makes it prohibitive for routine clinical use. An alternative is the use of grandparental samples for haplotype phasing [20,21]. While complexity and costs could prohibit this being developed for autosomal recessive conditions, it is more suitable for dominant disorders such as Rb, where only one set of grandparents would be required. We are therefore investigating this alternative phasing approach.

A further limitation of haplotype analysis is the small risk (1:500–1:1000) of recombination. Amplicon-based MPS has the advantage of directly testing the variant and so avoids this potential issue. For this reason and the smaller technical costs involved, amplicon-based MPS is preferable to capture-based targeted sequencing for paternal and likely de novo variants. However, the nature of cfDNA, which is naturally fragmented to approximately 140–160 bp in length, means amplicon-based NIPD testing is not suitable for larger paternal or de novo variants, such as indels over 10 bp. For families where the father is known to carry an *RB1* variant >10 bp, RHDO analysis is an option where the couple has had a previous child. We have already implemented RHDO for paternal inheritance in autosomal recessive disorders SMA and CF [7,12]. While haplotype analysis is not suitable for de novo variants, we report a proof-of-principle case where NIPD of a variant of ~2 Mb was achieved through capture-based targeted sequencing and direct detection. Based on the density of informative (Type 1) SNPs observed in Families L–M, we estimate that this analysis has the potential to detect deletions greater than 1 Mb. Although this detection would not possible where a variant resides on the maternal chromosome, it has been found that >90% of de novo *RB1* mutations are located on the paternal chromosome [22,23,24,25,26,27], therefore increasing the likelihood of assay success. However, due to the cost involved in this test and the low risk to the fetus, it is unlikely this will be implemented clinically until technology advances significantly reduce this cost.

A recent publication has confirmed that late diagnosis has a particularly detrimental impact on patients with Rb from low to middle-income countries (LMICs) [28]. One of the co-authors of the study previously identified a range of interventions that could impact on prognosis [29], which together with improved treatments in those countries will significantly increase the proportion of inherited cases in the next one to two decades. While our testing facility is based in the UK, this testing is available for international retinoblastoma patients as only blood samples are required, which can be stable to transport for up to five days’ post-blood draw if stored correctly. This could be of particular benefit to retinoblastoma patients within LMICs, where access to healthcare immediately after birth can be limited. In particular, NIPD would have significant advantages over transferring a newborn baby and early post-partum mother over large geographical distances to achieve early intervention if required. The feasibility of the transfer of DNA for ophthalmological genetic disorders to an international centre from a middle-income country has been reported by Zanolli et al. [30] and would likely reduce the need for patients with advanced Rb having to seek care on another continent [31].

In conclusion, NIPD of Rb presents a viable alternative to invasive prenatal and newborn genetic testing, offering many advantages for both the healthcare system and the family themselves, providing a safer and earlier diagnosis of their child.

## Figures and Tables

**Table 1:** Non-invasive prenatal diagnosis (NIPD) results for paternal and de novo *RB1* mutations by amplicon-based MPS. Read counts are given for both wild-type (WT) and mutant (MUT) alleles in each sample tested, with the overall percentage of mutant reads (Mut %) also shown. Maternal cfDNA results are the average of two duplicate samples. Cell-free DNA control results are the average reads from five plasma control cfDNA samples. Max mutant % refers to the highest mutant allele % across all five control plasma samples and denotes the background threshold. TBC indicates pregnancies ongoing.

Family	*RB1* Mutation	Basis of Test	Gestation (weeks)	Fetal Fraction (%)	Maternal cfDNA			Maternal gDNA			cfDNA Control				Paternal gDNA			Proband gDNA			Test Result	Result Confirmed
					WT	MUT	Mut %	WT	MUT	Mut %	WT	MUT	Mut %	Max Mut %	WT	MUT	Mut %	WT	MUT	Mut %		
A	c.1735C>T	Paternal	16	2.37	127,106	1622	1.26	155,826	241	0.15	109,510	164	0.15	0.21	80,200	81,497	50	NA			Mutation Present	Yes
B	c.1498+1G>C	Paternal	20	4.02	61,428	187	0.30	62,084	13	0.02	60,061	10	0.02	0.03	30,633	31,109	50	NA			Mutation Present	Yes
C	c.1072C>T	Paternal	12	13.5	140,559	266	0.19	162,249	264	0.16	133,482	244	0.18	0.25	71,898	68,025	49	NA			No Mutation Detected	Yes
D	c.1238A>T	Paternal	31	17.2	39,405	4888	11.03	60,681	25	0.04	57,778	50	0.09	0.17	50,872	53,671	51	NA			Mutation Present	Yes
E	c.1075A>T	Paternal	21	9.91	51,651	18	0.03	44,449	7	0.02	37,074	15	0.04	0.05	18,838	21,515	53	NA			No Mutation Detected	TBC
F	c.1039dupT	*De novo*	28	8.41	20,437	8	0.04	19,163	15	0.08	16,119	8	0.05	0.09	15,616	7	0.04	14,914	12,887	46	No Mutation Detected	Yes
G	c.1199dupT	*De novo*	20	10.4	74,901	23	0.03	82,034	21	0.03	74,392	29	0.04	0.06	71,395	35	0.05	69,513	61,300	47	No Mutation Detected	Yes
H	c.1333C>T	*De novo*	35	11.4	5713	21	0.37	6015	13	0.22	5148	18	0.35	0.55	5448	22	0.40	2668	2713	50	No Mutation Detected	Yes
I_1	c.1333C>T	*De novo*	12	7.99	25,603	43	0.17	23,721	23	0.10	22,731	40	0.18	0.23	21,609	36	0.17	18,880	19,798	51	No Mutation Detected	Yes
I_2	c.1333C>T	*De novo*	30	17.5	12,870	24	0.19	12,233	12	0.10	10,686	20	0.18	0.38	10,727	17	0.16	8519	8422	50	No Mutation Detected	Yes
J	c.1072C>T	*De novo*	16	8.52	585,218	2411	0.41	743,564	2649	0.35	641,821	2148	0.33	0.46	566,685	2077	0.37	608,198	521,483	46	No Mutation Detected	Yes
K	c958C>T	*De novo*	14	6.54	8925	9	0.10	8758	5	0.05	8481	11	0.13	0.16	7102	6	0.06	6487	6040	48	No Mutation Detected	TBC

## Supplementary Materials

The following are available online at https://www.mdpi.com/2077-0383/9/11/3517/s1, Table S1: Primer sequences for amplicon-based NIPD for Rb. Table S2: Primer sequences for amplicon-based fetal load quantification.
