# Supplementary material for: Non-Invasive Prenatal Diagnosis of Retinoblastoma Inheritance by Combined Targeted Sequencing Strategies
Source: J Clin Med. 2020 Oct 30;9(11):3517. doi: 10.3390/jcm9113517 (PMC7692133; doi:10.3390/jcm9113517)
Supplement: Supplementary file 1 [file jcm-09-03517-s001.pdf]

Supplementary Materials

Table S1. Primer sequences for amplicon based NIPD for Rb.

RB1 Mutation	Forward Primer	Reverse Primer	Family Tested
c.1735C>T	CATGTTTCATATAGGATTCACCTTTATTTG	AAGGTGATCAGTTGGTCCTTC	A
c.1498+1G>C	TGAGGTTGTAATGGCCACATATAG	ACCAAAGAAACACACCACATTT	B
c.1072C>T[1]	TTTGAAACACAGAGAACACCA	CTGGAGTGTGTGGAGGAATTA	C, J
c.1075A>T[1]			D
c.1238A>T	AGAACTGCACAGTGAATCCAA	CCACAGCTTTAGCAAATTTCTCTT	E
c.1039dupT	CTTTCTAAACGATACGAAGA	TGTGCAATACCTGTCTAT	F
c.1199dupT	TCAGCAAGTGATCAACCTTCA	CTTTGCCAAGATATTACAATAAATAATGTTTC	G
c.1333C>T	GATTTTCTAAAATAGCAGGCTCTTATTT	TACAAACTTGGAGTTCGCTTG	H, I
c.958C>T	TTCTTTCAAGGTTGAAAATCTTTCTAAA	TCCAAAAATAATCTTGCATCTAGATCT	K
c.1630delA	CAAAGCAGAAGGCAACTTGA	GGGATTCCATGATTCGATGTTC	NA

[1] One primer pair used for both mutations

Table S2. Primer sequences for amplicon based fetal load quantification.

SNP ID	Location (hg19)	Forward Primer	Reverse Primer
rs6968649	chr7: 16596685	TGGAGCAGAAGTAGAAGA	CTTGCTAGGCTTTCCAAA
rs131	chr7: 24938887	GTCTGGACAGAGCAGAAG	AATCCAGCAGTATTAACAGT
rs41342	chr7: 28709185	GTGGAGGGCATACAGGAA	GAAATAACTTCCGATTTTCAGAA
rs30560	chr7: 32062091	GGTAGATTTCAGACAAGTG	CATCACCCCATGATTTTC
rs38308	chr7: 70068401	GGACAGAGCCACACCTTT	GCATGTTCTAGACTGAAGCA
rs43005	chr7: 90763178	TACCAGATTCTCCTCTGTAGATC	ATGCCCTGTGGTTGTAAA
rs41264	chr7: 105616335	TGAGTCAGGGTTGCTTCC	CAAAGCTCTCCTAGTGACTAATG
rs193588	chr7: 116850770	TGAACCAAGCCTTGAAGAAGTAG	GCCCCACCACTTACCATTTCT
rs16806	chr8: 2963372	AATGGCTAGAAAGATAACAGATAG	GCTTCCGCTATGAAAACTT
rs10091913	chr8: 10853377	AATGTGAGAGGGCAATCC	GTCTGATTCAACTACAGGTG
rs354511	chr8: 15534444	AAACTGAAATAGCTAGAGC	GACTTTCAATATATACAACCATC
rs97635	chr8: 15608681	AGTTTGGGTTGCTCTGTT	CCATTACCTAAAATACCCTGATTG
rs967317	chr8: 59972309	TTATTCCCTACTTGATTTCCCTTAT	TGAAACAATGCCACAAAGC
rs13279476	chr8: 73821487	CACATTGGTGTAGCTCAT	CTCATTGGGTTGACATCT
rs16884	chr8: 97875315	ACTACCTTGTTGAAGCCTCT	TGAATTGGTTAGAATCAGATTGTAAGA
rs2705274	chr8: 138959247	TCAGAAAGTGTTTTGGAGTAAAGTA	ACAATCCACAGGGGAAAGA
rs1926394	chr9: 7315722	GGAGAGGGTGAGTGTTCA	AATTATAGATTAGCTTCAAGATGGC
rs1339143	chr9: 23218974	GGAAACAATCGTGGTAAG	GTTTCTTGGACTCTATCAG
rs2938882	chr9: 81187857	TTGCCATTGTCTTCAACC	CTTTGGTTAAGGATTTCTGAC
rs1929709	chr9: 83752537	TTATAAGACTCATGTTGACTGTATAG	GAGCCATTTGCCCTTTTC
rs191623	chr9: 89098238	TGAAATGAGCATGGCATAGTATT	TGCATGGCACCCAGAATC
rs2025151	chr9: 99161512	CTTTCGGCTCAGCACAAG	GGACACTGTGGTTGCTTAG
rs182705	chr9: 102085812	CCAACAGGAAGCAATAGA	AGCCCAACTTATAGAATTTC
rs1809924	chr9: 107814469	GTGGGGCTAGGATGATTAGAAT	TGTGGGTCCTGTGCATTA
rs2797820	chr9: 136557606	GCAGGTCACACTGGTTCT	GAAGGATGGGGAGGAGCC
rs7489602	chr13: 23994932	TGATGTTCATCAGGAGCC	ACACTTGACTCCAAATGTCT
rs2274276	chr13: 50095953	TTTCTATTCAATGAACCCTCTC	GCAAAACAGGTAAATAATTGAGTA
rs339528	chr13: 60562931	ATATCAGAAATTATCTCCCATGCG	CACCAAATAATTAAGGCTTACCAT
rs1535802	chr13: 74318497	CAAAATGGGGTCAATACTACCTA	CCTTTCATCCATGCCCTC
rs342718	chr13: 92392966	TTAAACAGCCAGCAAAGC	GAGCGCAATGTCCTTTAC
rs9585307	chr13: 100621776	AAGAAGGTGTGCGTGAAG	AAGACAGGAATGCGCTTTC
rs4405424	chr13: 108330594	TTCCTTCCAATGAACTCCACA	CCATACGCTGAGCACACA
rs831150	chr13: 111773316	TGCCACCTATACTGAACTT	CCGTCTCCTTTCCTGATC
rs8085897	chr18: 361365	CTCTCTAGTCATCGTTTC	ATGAAAAGATCTCACACTA
rs1784762	chr18: 2895959	TAAGCAGATCGCCAGACC	TGAAGAGGGCAACAGGCT
rs569589	chr18: 3555986	TATCTTGAAACTTCTTTCTTTAGAGAG	ATATACAATGCTCTGCGTGTT
rs588257	chr18: 11992479	AGGAGCACAAGAACAAGA	GAGACATCCTAGGCTAACATA
rs1941702	chr18: 31236980	AACATTAATAGATCGACTGCTCTG	CCACAGGCTTGGAAACTC
rs4245250	chr18: 45588281	ACTTTGAAATAGTCAGTATCACTTCTA	TGCCATTCTTTATTACTGATGCT
rs1790580	chr18: 67465595	GGTAGGAGGGCAAAGTTC	TTCAGCGTTTGTAACTCTT
rs1810181	chr18: 77072267	GATTGGGTGGTGCCTTCTA	ACACAGTAATATAACTGCATTTAGGAA
SRY	chrY:2655608-2655670	TTGTCGCACTCTCCTTGT	TGAATACGCTTAACATAGCAGAAG
